# Regulatory effects of tea polysaccharides on hepatic inflammation, gut microbiota dysbiosis, and serum metabolomic signatures in beef cattle under heat stress

**DOI:** 10.3389/fphys.2024.1460414

**Published:** 2024-09-06

**Authors:** Fan Li, Jun Xu, Min Xie, Dan Fei, Yaomin Zhou, Xiong Li, Yelan Guang, Lihui Gong, Lizhen Hu, Fan Feng

**Affiliations:** ^1^ Institute of Animal Husbandry and Veterinary Science, Jiangxi Academy of Agricultural Sciences, Nanchang, China; ^2^ Jiangxi Province Key Laboratory of Animal Green and Healthy Breeding, Nanchang, China; ^3^ Institute of Quality Safety and Standards of agricultural Products, Jiangxi Academy of Agricultural Sciences, Nanchang, China; ^4^ Pingxiang Center of Agricultural Science and Technology Research, Pingxiang, China

**Keywords:** tea polysaccharides, inflammation, long-chain fatty acids, gut micobiota, liver

## Abstract

**Background:**

Long-term heat stress (HS) severely restricts the growth performance of beef cattle and causes various health problems. The gut microbiota plays a crucial role in HS-associated inflammation and immune stress involving lymphocyte function. This study investigated the effects of dietary tea polysaccharide (TPS), a natural acidic glycoprotein, on HS-induced anorexia, inflammation, and gut microbiota dysbiosis in Simmental beef cattle.

**Methods:**

The cattle were divided into two groups, receiving either normal chow or normal chow plus TPS (8 g/kg, 0.8%). Transcriptome sequencing analysis was used to analysis the differential signaling pathway of liver tissue. 16S rDNA sequencing was performed to analysis gut microbiota of beef cattle. Serum metabolite components were detected by untargeted metabolomics analysis.

**Results:**

Hepatic transcriptomics analysis revealed that differentially expressed genes in TPS-fed cattle were primarily enriched in immune processes and lymphocyte activation. TPS administration significantly reduced the expression of the TLR4/NF-κB inflammatory signaling pathway, alleviating HS-induced hepatic inflammation. Gut microbiota analysis revealed that TPS improved intestinal homeostasis in HS-affected cattle by increasing bacterial diversity and increasing the relative abundances of *Akkermansia* and *Alistipes* while decreasing the *Firmicutes*-to-*Bacteroidetes* ratio and the abundance of *Agathobacter*. Liquid chromatography-tandem mass spectrometry (LC‒MS/MS) analysis indicated that TPS significantly increased the levels of long-chain fatty acids, including stearic acid, linolenic acid, arachidonic acid, and adrenic acid, in the serum of cattle.

**Conclusion:**

These findings suggest that long-term consumption of tea polysaccharides can ameliorate heat stress-induced hepatic inflammation and gut microbiota dysbiosis in beef cattle, suggesting a possible liver-gut axis mechanism to mitigate heat stress.

## 1 Introduction

Heat stress refers to a series of physiological reactions that occur in animals when they are exposed to environmental conditions that exceed their thermal tolerance (Giannone et al., 2023). Beef cattle are particularly susceptible to heat stress in hot and humid environments due to their high basal metabolism, rapid growth, efficient heat conservation, and limited sweating capacity (Thornton et al., 2022; Carvajal et al., 2021). Recent studies have shown that heat stress adversely affects feed intake, disease resistance, growth and development, and meat quality in cattle (Chen et al., 2023; Vijayakumar et al., 2022; Li et al., 2020; Zhang et al., 2020).

Inflammation is the body’s initial response to external damage or invasion and is aimed at eliminating the source of injury and initiating tissue repair through the activation and recruitment of immune cells (Medzhitov, 2008). There is increasing evidence suggesting that heat stress can induce the upregulation of inflammatory cytokines and immune cells, including granulocytes, lymphocytes, monocytes, and macrophages (Min et al., 2016). The liver, as the most metabolically active organ, is known to suffer functional impairments under conditions of heat stress in various animal models. For example, heat stress can result in the disruption of liver lobular structure, diffuse necrosis, and hemorrhage in both C57BL/J mice and broilers (Liu Y. L. et al., 2022; Lan et al., 2023; Chen et al., 2020). Additionally, heat stress can significantly increase the expression of inflammatory factors such as IL-6, TNF-α, NF-κB p65, and IκBα and increase the levels of TNF-α, IL-1β, and IL-6 (Mohyuddin et al., 2021). Therefore, the prevention and treatment of heat stress have significant implications for mitigating immune, metabolic, and reproductive disorders associated with heat stress in beef cattle.

Tea polysaccharide (TPS) is a naturally occurring acidic glycoprotein with a variety of biological activities found in tea. It is composed of different monosaccharides connected by glycosidic bonds, as well as proteins and minerals. Recent research highlights the significant physiological role of tea polysaccharides in enhancing immune function, the inflammatory response, and intestinal health (Luo et al., 2023; Chen et al., 2019). Chen et al. demonstrated that feeding tea polysaccharides to broilers increased the serum immunoglobulin IgG content, T-lymphocyte count, and lymphocyte conversion rate (Chen et al., 2021). Similarly, Deng et al. reported that the oral administration of tea polysaccharides to mice significantly reduced the levels of proinflammatory cytokines (TNF-α and IL-1β) and increased the levels of anti-inflammatory cytokines (IgA, IgG, IgM, IL-4, and IL-10) (Deng et al., 2023). Moreover, other studies have shown that tea polysaccharides have a protective effect on acute and chronic liver injury. Feeding mice tea polysaccharides notably alleviated acute liver injury induced by carbon tetrachloride and high sugar while decreasing the serum levels of ALT and AST enzymes and increasing the activities of superoxide dismutase (SOD) and glutathione peroxidase (GSH-Px) in the liver (Zhai et al., 2017). However, the potential of tea polysaccharides to alleviate heat stress-related hepatic inflammation and impairment in beef cattle remains to be investigated.

The gut microbiota is a vital component of the body’s microbial system and plays a significant role in regulating various physiological processes, such as obesity, neurotransmission, intestinal inflammation, diabetes, aging, and coronary artery disease (de Vos et al., 2022; Yoo et al., 2020). Recently, there has been considerable interest in the impact of the gut microbiota on hepatic function. Research has indicated that the dysregulation of the intestinal microbiota induced by lipopolysaccharide (LPS) and a high-fat diet can activate the TLR4/NF-κB inflammatory signaling pathway in the liver (Kang et al., 2023; Liu et al., 2022b). Moreover, alcoholic liver disease and fatty liver disease are associated with an abnormal gut microbiota structure characterized by a greater ratio of *Firmicutes-to-Bacteroidetes* than in normal mice (Zhang et al., 2023a; Aragon-Vela et al., 2021). Notably, several studies have explored the regulatory effect of tea polysaccharides on the intestinal microbiota. Zhu et al. reported that tea polysaccharide improved intestinal barrier function and alleviated gut microbiota dysbiosis in high-fat diet-fed rats by increasing the abundance of beneficial bacteria such as *Akkermansia, Bacteroides*, and *Desulfovibrio* (Zhu et al., 2023). Similarly, Du et al. demonstrated that tea polysaccharide reversed gut dysbiosis by enriching beneficial bacteria such as *Lactobacillus*, *Parabacteroides*, *Akkermansia, Bifidobacterium, and Roseburia* (Du et al., 2022). Importantly, *Akkermansia*, a Gram-negative bacterium, has been found to play a crucial role in combating metabolic diseases such as obesity and diabetes (Depommier et al., 2019). Studies have shown that the addition of *Akkermansia* or polyphenols from table grapes can significantly increase the abundance of intestinal *Akkermansia* and ameliorate hepatic inflammation in obese or diabetic mice (Zhao et al., 2017; Tu et al., 2018). These findings highlight the significant role of the intestinal microbiota in regulating liver function and suggest a potential mechanism by which tea polysaccharides may mitigate liver inflammation. However, the extent to which tea polysaccharides can alter the gut microbiota and alleviate hepatic inflammation in beef cattle under heat stress remains largely unknown.

In the present study, we investigated the regulatory effects of tea polysaccharides on hepatic inflammation, gut microbiota dysbiosis, and serum metabolomic signatures in beef cattle under heat stress. Hepatic transcriptomics analysis demonstrated that tea polysaccharides could alleviate immune and inflammatory responses in cattle liver under heat stress and significantly decrease the expression of the TLR4/NF-κB inflammatory signaling pathway. Furthermore, 16S rRNA sequencing analysis indicated that tea polysaccharide improved gut microbiota dysbiosis by increasing the relative abundances of beneficial bacteria such as *Akkermansia* and *Alistipes* while decreasing the *Firmicutes*-to-*Bacteroidetes* ratio and the abundance of *Agathobacter*. *Akkermansia* and *Alistipes* have been reported to be closely related to fatty acid metabolism and the regulation of body inflammation. Consistently, our research showed that tea polysaccharides significantly increased the serum levels of long-chain fatty acids, including stearic acid, linolenic acid, arachidonic acid, and adrenic acid, all of which have been verified to have anti-inflammatory functions in the body (Ma et al., 2019). Furthermore, network analysis of the microbiota and metabolites confirmed the correlation between the gut microbiota and serum fatty acid levels. Overall, our study suggested that long-term consumption of tea polysaccharides can ameliorate heat stress-induced hepatic inflammation and gut microbiota dysbiosis in beef cattle, suggesting a possible liver-gut axis mechanism to mitigate heat stress. These findings provide a reference for the potential application of tea polysaccharides in alleviating heat stress in beef cattle.

## 2 Materials and methods

### 2.1 Animal experimentation

Twelve healthy male Simmental beef cattle (500 kg ± 20 kg) in the fattening period were purchased from Jiangxi Shanggao Lifeng Pastoral Industry Co., Ltd. The beef cattle were divided into two groups: the control group and the TPS group (Tea polysaccharide purchased from Hunan Century Huaxing Biotechnology Co., Ltd., food grade, purity 99%). Each group contained six replicates, with one cattle per replicate. The heat stress model was created by feeding the cattle high temperature and humidity weather conditions from June to August in Jiangxi summer. A temperature and humidity meter was hung in the middle and end of the test barn about 1.5m from the ground. During the official period, the temperature and relative humidity were recorded every day to calculate the barn THI (THI>75, calculated using the following formula: THI = 0.81 × T + RH ×(T-14.40)+46.40, where THI is temperature and humidity index, T is the ambient temperature in Celsius and RH is the relative humidity).The THI are shown in Table 1. The control group was fed a regular basic diet, while the TPS group was fed a basic diet supplemented with 0.8% tea polysaccharide (based on dry matter, dosage was selected with reference to literature (Zhong et al., 2015; Zhong et al., 2014) and company recommendation),The composition and nutrient level of the experimental diet are shown in Table 2. All experimental feed was thoroughly mixed and made uniform using a TMR feed mixer truck, and the experiment lasted for a total of 56 days (including a 6-day prefeeding period and a 50-day formal trial period). The experimental cattle were raised in pens according to the number of repetitions and allowed to drink freely. The physiological condition and feed intake of the cattles were monitored at 6:00 and 18:00 every day.

**TABLE 1:** The barn temperature and humidity index.

Item	Temperature	Humidity	THI	Heat stress intensity
08:00a.m.	32.11 ± 0.82	58.11 ± 1.74	82.7 ± 1.13	Moderate
14:00p.m.	34.08 ± 1.43	53.71 ± 0.88	84.58 ± 1.92	severe
20:00p.m.	29.32 ± 1.29	72.99 ± 5.98	80.97 ± 1.36	Moderate

**TABLE 2:** Composition and nutrient level of basic diet.

Ingredients	Content (%)	Nutrient level	Content (%)
Peanut seedling	45.00	Dry matter DM	89.91
Brewer’s grains	5.00	Net energy NE (MJ/kg)	7.03
Corn	29.00	Crude protein CP	12.45
Soybean meal	2.00	Neutral detergent fibre NDF	34.14
Cottonseed meal	4.50	Acid detergent fibre ADF	25.53
Wheat bran	11.00	Ash	9.89
Limestone	0.60	No nitrogen extract NFE	52.63
NaHCO_3_	0.50	Calcium Ca	0.69
CaHPO_4_	0.70	Phosphorus P	0.30
NaCl	0.70		
Premix	1.00		
Total count	100.00		

Premix is provided for each kilogram of diet VA 20 000 IU, VD3 30,000 IU, VE 1 500 IU, Cu 500 mg, Fe 100 mg, Mn 60 mg, Zn 60 mg, Se 50 mg, I 0.5 mg.

### 2.2 Analysis of the gut microbiota by 16S rDNA sequencing

The gut microbiota was characterized by sequencing the 16S rRNA/ITS/18S rRNA region of all bacteria in the fecal samples using the MiSeq Illumina Sequencing Platform (NovaSeq 6000, Illumina, United States). Fresh fecal samples were collected from four individual cattle in each group, and the total genomic DNA of each sample was extracted using HiPure Stool DNA Kits (Magen, D3141). After confirming the quality of the DNA samples, 16S rDNA sequencing was performed by Genedenovo Biotechnology Company (Guangzhou, China).

### 2.3 Bioinformatics data analysis of 16S rDNA sequencing

After filtration and merging of the raw data, the operational taxonomic units (OTUs) were clustered and annotated using UPARSE with a similarity cutoff of 97%. α diversity, including principal component analysis (PCA), the Chao1 index, the PD-tree index, the ACE index, the Simpson index, and the Shannon index, was analyzed using Mothur. β diversity analyses, including unweighted pair group method with principal coordinate analysis (PCoA), PCA, and nonmetric multidimensional scaling (NMDS), were performed using the QIIME software package. The linear discriminant analysis (LDA) effect size (LEfSe) algorithm was used to identify differences in microbial compositions between groups and biomarker species in different groups.

### 2.4 Untargeted metabolomics analysis

#### 2.4.1 Metabolite extraction

Before morning feeding on the first day of the last week of the formal period, blood was collected from the jugular vein of beef cattle using vacuum blood collection vessels, and was placed in a centrifuge at 3,000 r/min for 15 min after resting for 30min. The separated serum was refrigerated at −80°C for later use.Then the serum samples (100 μL) were placed in the EP tubes and resuspended with 400 μL prechilled 80% methanol by well vortex. Then the samples were incubated on ice for 5 min and centrifuged at 15,000 g, 4°C for 20 min. Some of supernatant was diluted to final concentration containing 53% methanol by LC-MS grade water. The samples were subsequently transferred to a fresh Eppendorf tube and then were centrifuged at 15,000 *g*, 4°C for 20 min. Finally, the supernatant was injected into the LC-MS/MS system analysis.

#### 2.4.2 UHPLC-MS/MS analysis

UHPLC-MS/MS analyses were performed using a Vanquish UHPLC system (Thermo Fisher, Germany) coupled with an Orbitrap Q ExactiveTM HF-X mass spectrometer (Thermo Fisher, Germany) at Gene Denovo Co., Ltd. (Guangzhou, China). The samples were injected onto a Hypesil Gold column (100 × 2.1 mm, 1.9 μm) using a 17-min linear gradient at a flow rate of 0.2 mL/min. The eluents for the positive polarity mode were eluent A (0.1% FA in water) and eluent B (methanol). The eluents for the negative polarity mode were eluent A (5 mM ammonium acetate, pH 9.0) and eluent B (methanol). The solvent gradient was set as follows: 2% B, 1.5 min; 2%–100% B, 12.0 min; 100% B, 14.0 min; 100%–2% B, 14.1 min; and 2% B, 17 min. The Q ExactiveTM HF-X mass spectrometer was operated in positive/negative polarity mode with a spray voltage of 3.2 kV, a capillary temperature of 320°C, a sheath gas flow rate of 40 arb, and an aux gas flow rate of 10 arb.

#### 2.4.3 Data processing and metabolite identification

The raw data files generated by UHPLC-MS/MS were processed using Compound Discoverer 3.1 (CD3.1, Thermo Fisher) for peak alignment, peak picking, and quantitation of each metabolite. The main parameters were set as follows: retention time tolerance of 0.2 min, actual mass tolerance of 5 ppm, signal intensity tolerance of 30%, signal/noise ratio of 3, and minimum intensity of 100,000. The peak intensities were subsequently normalized to the total spectral intensity. The normalized data were then used to predict the molecular formula based on additive ions, molecular ion peaks, and fragment ions. Peaks were matched with the mzCloud (https://www.mzcloud.org/), mzVault, and Mass List databases to obtain accurate qualitative and relative quantitative results. Statistical analyses were performed using R software (version R-3.4.3), Python (version 2.7.6), and CentOS (release 6.6). In cases where the data were not normally distributed, attempts were made to normalize them using the area normalization method.

### 2.5 Liver transcriptome sequencing analysis

At the end of the experiment, the experimental beef cattle were slaughtered, and the liver tissues were collected and frozen at −80°C for testing. Similar to the samples used for metabolomics analysis, four freeze-dried liver samples were used for transcriptomics analysis. Total RNA was extracted from each sample using a TRIzol reagent kit (Life Technologies). RNA quality was assessed on an Agilent 2100 Bioanalyzer (Agilent Technologies, Palo Alto, CA, United States) and checked using RNase-free agarose gel electrophoresis. After extraction of total RNA, eukaryotic mRNA was enriched using mRNA Capture Beads with the Hieff NGS^®^ Ultima Dual-mode mRNA Library Prep Kit (12309 ES, Yeasen). The enriched mRNA was then fragmented into short fragments using fragmentation buffer and reverse transcribed into cDNA with random primers. After RNA quality assessment, mRNA purification, and fragmentation, transcriptome sequencing analysis was conducted by Genedenovo Biotechnology Company (Guangzhou, China).

### 2.6 Statistical analysis

All values in this experiment are reported as the mean ± standard error (SEM), and comparisons between two groups were performed using Student’s t-test. The criteria for significant differences were set as *P< 0.05, **P< 0.01, and ***P< 0.001. The statistical analysis and data visualization were performed using GraphPad Prism 8.0 software.

## 3 Results

### 3.1 Effects of tea polysaccharides on hepatic inflammation and immune stress in beef cattle under heat stress

**FIGURE 1:**
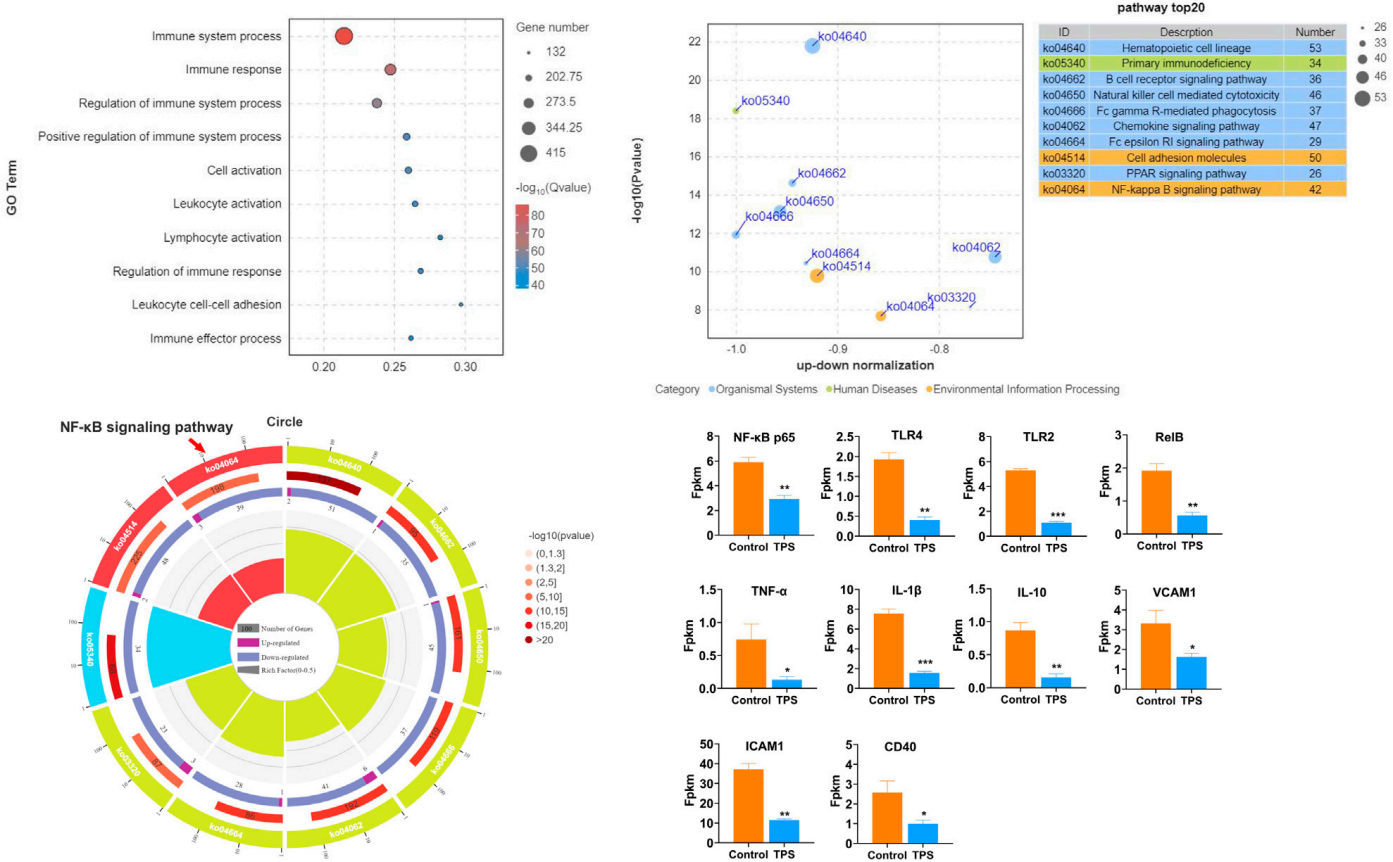
Effects of tea polysaccharides on hepatic inflammation and immune stress in beef cattle under heat stress. **(A)**. Gene Ontology (GO) functional enrichment analysis of the top 10 differentially expressed genes (DEGs) in the liver of the control and tea polysaccharide groups. **(B)**. Kyoto Encyclopedia of Genes and Genomes (KEGG) pathway enrichment analysis of the top 10 DEGs in the livers of the control and tea polysaccharide groups. **(C)** The top 10 GO enrichment circles of DEGs in the liver of the control and tea polysaccharide groups. **(D)** The map showing the relative expression levels of genes involved in inflammation in the liver of cattle. *p < 0.05, **p < 0.01 and ***p < 0.001, n = 4.

To examine the influence of tea polysaccharides on heat stress-induced liver dysfunction and damage, we initially established a model of heat stress stimulation in beef cattle. We then compared two groups: a control group fed normal chow and a group fed normal chow supplemented with tea polysaccharides (8 g/kg, 0.8%). Fig. *Sp1* illustrates the feed intake of the beef cattle during the experiment. As shown in Fig. *Sp1*, dietary supplementation with 0.8% tea polysaccharide resulted in a significant increase in the feed intake of beef cattle. This observation suggested that feeding tea polysaccharides could improve the physiological state and alleviate feeding inhibition under heat stress (FIG. Sp1A). The PCA plot shows a significant difference in transcription levels between the two groups (FIG. Sp1B). The results of GO functional enrichment analysis of the beef liver transcriptome revealed that after tea polysaccharide feeding, the differentially expressed biological processes were primarily associated with immune system regulation, immune response, leukocyte adhesion, and immune cell activation (Figure 1A). Furthermore, KEGG pathway enrichment analysis demonstrated that the differentially expressed genes were mainly enriched in signaling pathways such as primary immunodeficiency, B lymphocyte receptor, chemokine, Fc-γR, FC-Epsilon RI, and TLR4/NF-κB (Figure 1B). Additionally, enrichment circle analysis revealed significant changes in the TLR4/NF-κB pathway, as indicated by decreases in the expression of inflammatory marker genes such as NF-κB p65, TLR2, TLR4, RelB, TNF-α, and IL-1β (Figures 1C, D). To summarize, these results indicate that tea polysaccharides can effectively alleviate inflammatory and immune responses in beef cattle liver under heat stress, potentially through the TLR4/NF-κB inflammatory signaling pathway.

### 3.2 Tea polysaccharides improved the heat stress-induced decrease in the richness and diversity of the gut microbiota in beef cattle

**FIGURE 2:**
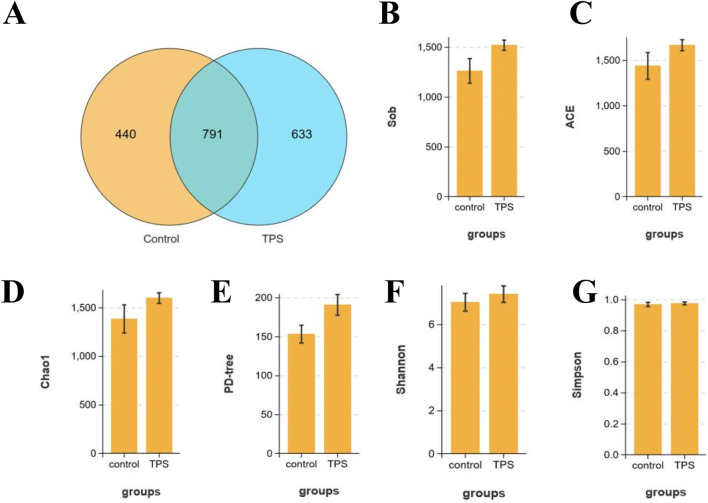
Effects of tea polysaccharides on microbiota composition of beef cattle under heat stress. **(A)** Stachyose increased the alpha diversity of the gut microbiota in beef cattle. Venn diagram showing the number of the same microbiota and unique microbiota between the control and tea polysaccharide groups (OTU level). **(B–G)** The observed species **(B)**, ACE index **(C)**, Chao1 index **(D)**, PD-tree index **(E)**, Simpson index **(F)** and Shannon index **(G)** were used to describe the alpha diversity of bacterial assemblages in beef cattle between groups. n = 4.

To further investigate the effect of tea polysaccharides on gut microbiota dysbiosis in beef cattle under heat stress, 16S RNA sequencing was conducted on the gut microbiota. OTU Wayne analysis was utilized to evaluate the variations in the gut microbiota structure (Figure 2A). Figure 2A shows significant changes in the microbiota composition of beef cattle in the tea polysaccharide-fed group compared to that in the control group. The positive effect of tea polysaccharides on enhancing the species richness of the microbiota was further supported by alpha diversity estimates. The results demonstrated that tea polysaccharides noticeably increased the number of observed species in the intestinal microbiota of beef cattle (Figure 2B). Specifically, the ACE, Chao 1, and PD-tree indices increased following tea polysaccharide intervention, indicating improved gut microbiota diversity (Figures 2C–E). However, there were no significant alterations in the Simpson and Shannon indices (Figures 2F, G). Thus, the results indicate that tea polysaccharide intervention significantly improved the reduction in species richness induced by heat stress but had no impact on the diversity of the flora.

**FIGURE 3:**
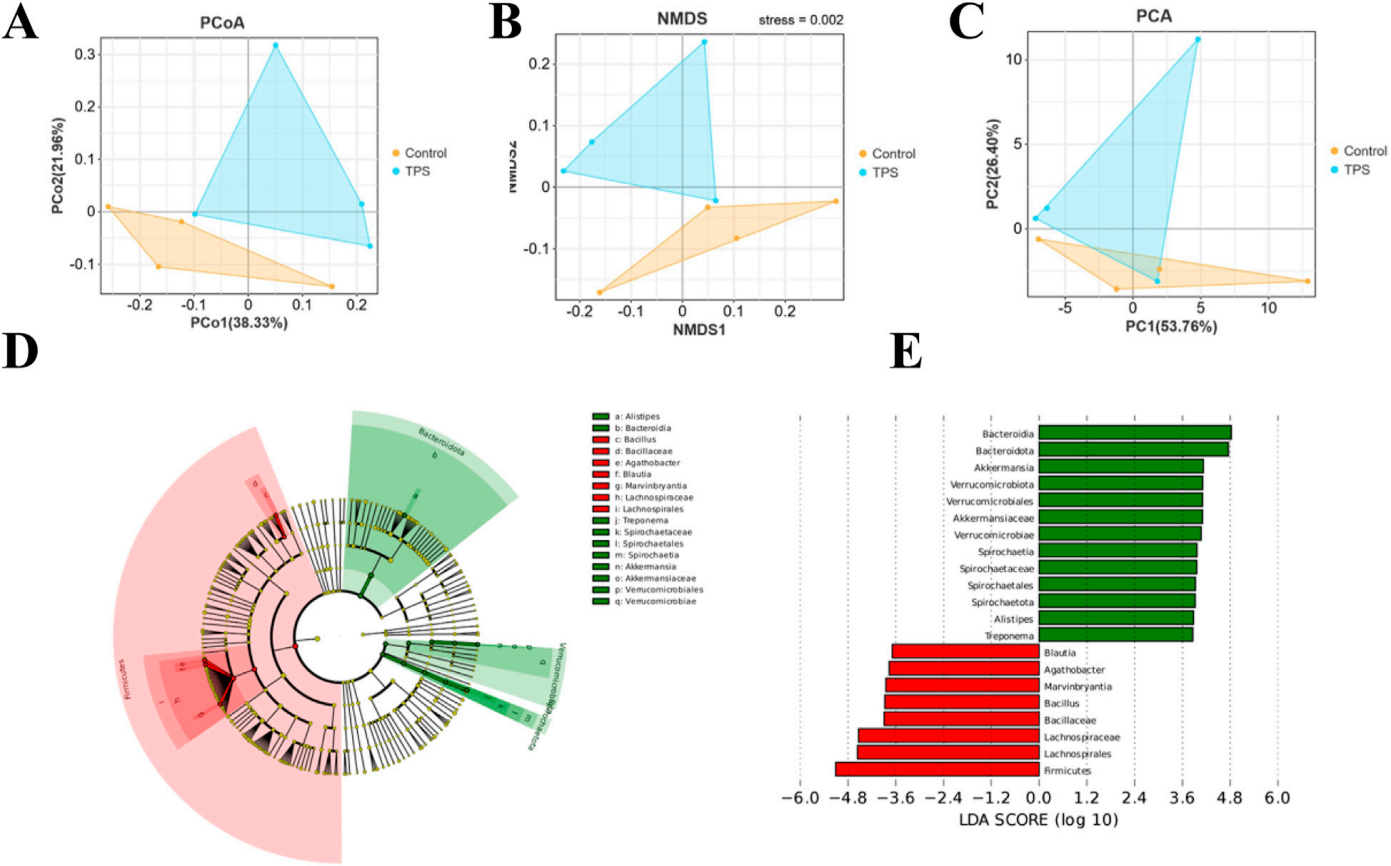
Bacterial community composition similarity. Tea polysaccharides improved the effects of heat stress on the bacterial community. **(A)** Principal coordinate analysis (PCoA) plots. **(B)** Nonmetric multidimensional scaling (NMDS) plots. **(C)** Principal component analysis (PCA) based on Bray−Curtis dissimilarity. **(D)** Cladogram visualizing the output of the LEfSe algorithm, which identifies taxonomically consistent differences between groups. The yellow dots represent nonsignificant bacteria in the groups; other colored dots are significant bacteria in the group labeled with the same color. **(E)** Histogram showing the LDA scores computed for features (genus level) that were differentially abundant between different treatments. The higher the LDA score is, the more significant the bacteria are.

Beta diversity analyses were then performed to compare the overall microbial profiles of cattle receiving different treatments, as shown in Figure 3A−C. The PCoA, NMDS, and PCA results revealed distinct microbial compositions in the groups of cattle treated with tea polysaccharides compared to those in the control group (Figures 3A–C). To identify key phylotypes significantly influenced by tea polysaccharides, all effective sequences from the samples were analyzed using the LEfSe method. Figure 3D, E depict the enrichment of the *Akkermansiaceae* family and *Akkermansia* genus, *Rikenellaceae* family and *Alistipes* genus, and *Spirochaetaceae* family and *Treponema* genus in the tea polysaccharide group. Conversely, the control group showed enrichment of the *Lachnospiraceae* family and *Agathobacter* genus, *Lachnospiraceae* family and *Marvinbryantia* genus, *Lachnospiraceae* family and *Blautia* genus, and *Bacillaceae* family and *Bacillus* genus (Figures 3D, E). Overall, these results indicate that tea polysaccharides reshape the structural composition of the gut microbiota in beef cattle under heat stress.

### 3.3 Changes in the taxonomic composition of the cecum microbiota communities at the phylum and genus levels

**FIGURE 4:**
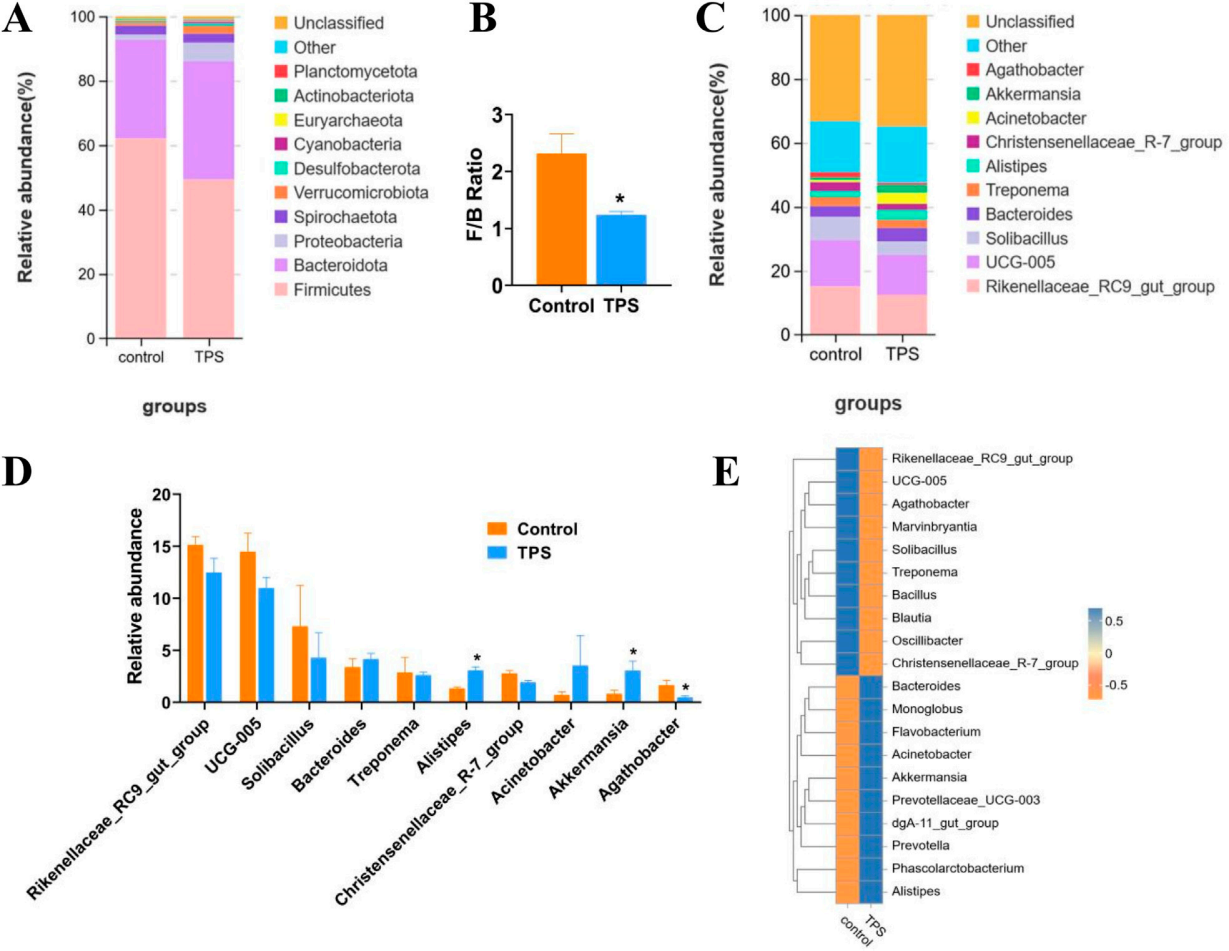
Changes in the taxonomic composition of the cecum microbiota communities at the phylum and genus levels **(A–C)** Changes in the relative abundance of bacteria at the phylum and genus levels. **(B)** The *Firmicutes*-to-*Bacteroidetes* ratio of the groups. **(D)** Bacteria with significant changes in abundance (genus level). **(E)** Species abundance heatmap between the microbiota and samples of the groups at the genus level. *p < 0.05, n = 4.

The bacterial community composition in different cattle groups was evaluated by examining the degree of taxonomic similarity at the phylum and genus levels. At the phylum level, the abundances of *Firmicutes* and Actinobacteria significantly decreased following tea polysaccharide supplementation, while the abundances of *Bacteroidetes*, Bacillobacteria, and Verrucoides significantly increased (Figure 4A). Moreover, the *Firmicutes*-to-*Bacteroidetes* ratio (F/B ratio) in the tea polysaccharide group was significantly lower than that in the control group (Figure 4B). At the genus level, the abundances of three bacterial species exhibited significant changes in response to tea polysaccharides. Specifically, tea polysaccharides substantially increased the abundance of *Akkermansia* and *Alistipes* while decreasing the abundance of *Agathobacter* (Figures 4C, D). The heatmap in Figure 4E illustrates the correlation between the bacterial communities at the genus level and the different samples. Among the three bacteria shown in Figure 4D, *Akkermansia* and *Alistipes* exhibited a strong correlation with the tea polysaccharide group, while *Agathobacter* was strongly associated with the control group (Figure 4E). Overall, these results indicate that tea polysaccharides have a significant impact on the microbiota structure of cecum contents in beef cattle following treatment, suggesting that intestinal microorganisms may represent a potential mechanism for the regulation of body function in beef cattle.

### 3.4 Influence of tea polysaccharides on the serum metabolomic signatures of beef cattle

**FIGURE 5:**
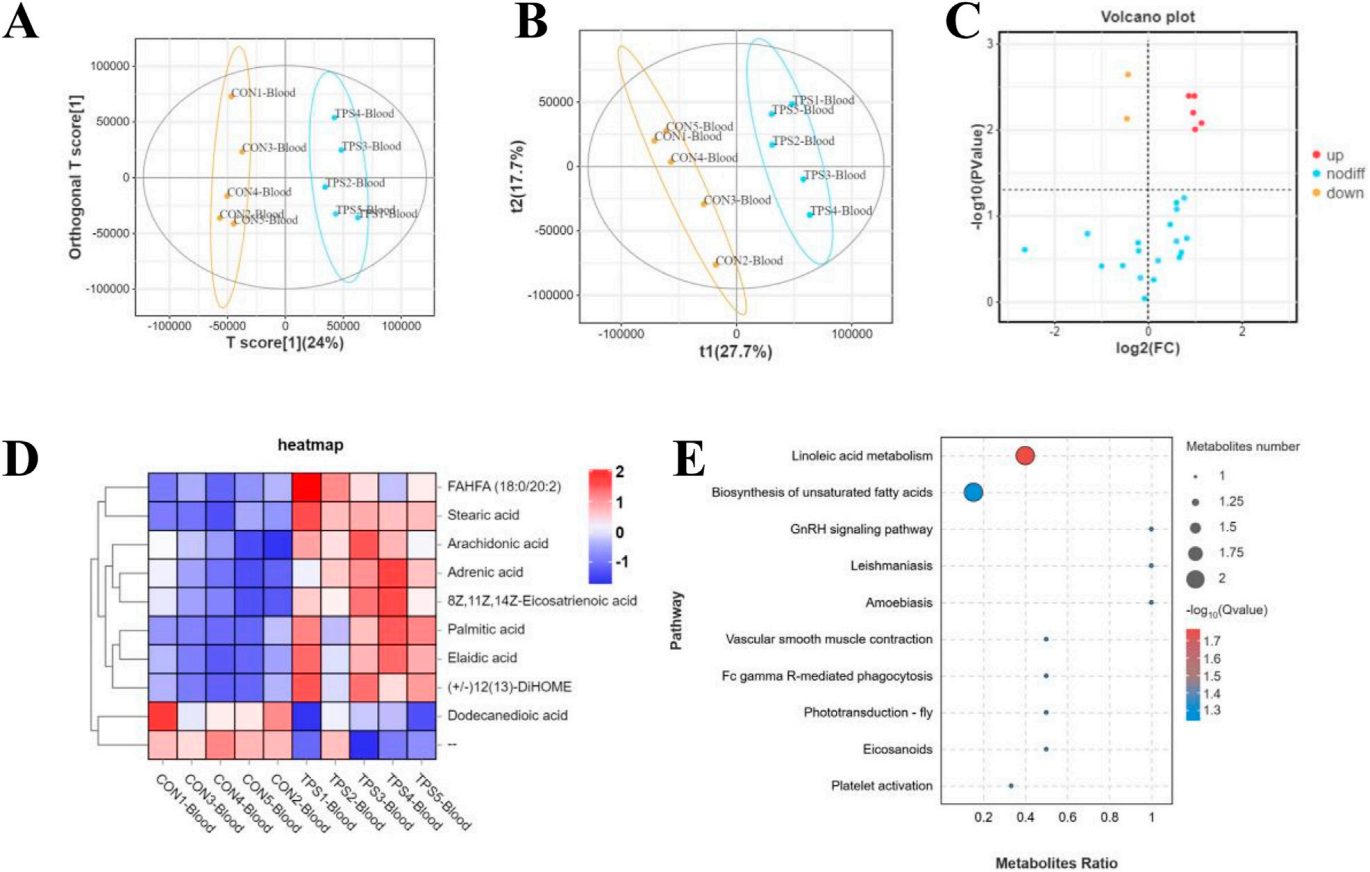
Influence of tea polysaccharides on the serum metabolomic signatures of beef cattle. **(A)** Plots of orthogonal partial least squares-discriminant analysis (OPLS-DA). **(B)** Partial least squares discriminant analysis (PLS-DA) scaling plots. **(C)** Volcano plot showing changes in the relative abundance of metabolites in the groups. **(D)** Heatmap of differentially abundant metabolites of the groups. **(E)** KEGG pathway enrichment analysis of differentially abundant metabolites in the serum of the control and tea polysaccharide groups. N = 5.

Furthermore, to investigate the potential mechanism by which tea polysaccharides affect liver function in beef cattle, nontargeted metabolomics detection was conducted on beef serum after tea polysaccharide feeding. The results of OPLS-DA and PLS-DA revealed significant differences in the serum levels of related metabolites between the groups (Figures 5A, B). Long-chain fatty acids (LCFAs) play a crucial role as nutrients and energy sources in the animal body. They are widely distributed in various tissues, organs, and blood. Previous studies have shown that long-chain fatty acids have diverse physiological functions, including promoting energy metabolism, maintaining intestinal barrier function, and combating inflammation. Consistently, the volcano map and cluster heatmap analyses indicated a significant increase in the serum levels of stearic acid, linolenic acid, arachidonic acid, and adrenic acid in the tea polysaccharide feeding group, all of which have established anti-inflammatory effects. This finding further supports the notion that tea polysaccharide feeding can considerably improve the level of inflammation in beef cattle and alleviate its occurrence (Figures 5C, D). Additionally, KEGG pathway enrichment analysis was performed to corroborate these differentially abundant metabolites according to the KEGG database. The analysis revealed that the metabolites were primarily enriched in the linoleic acid metabolism and unsaturated fatty acid synthesis pathways, consistent with the serum metabolomics findings (Figure 5E). Therefore, these results demonstrate that tea polysaccharides have a significant impact on the levels of serum long-chain fatty acids and may be involved in alleviating inflammation under heat stress.

### 3.5 Association analysis of intestinal microbiota and serum metabolites in tea polysaccharide-fed beef cattle

**FIGURE 6:**
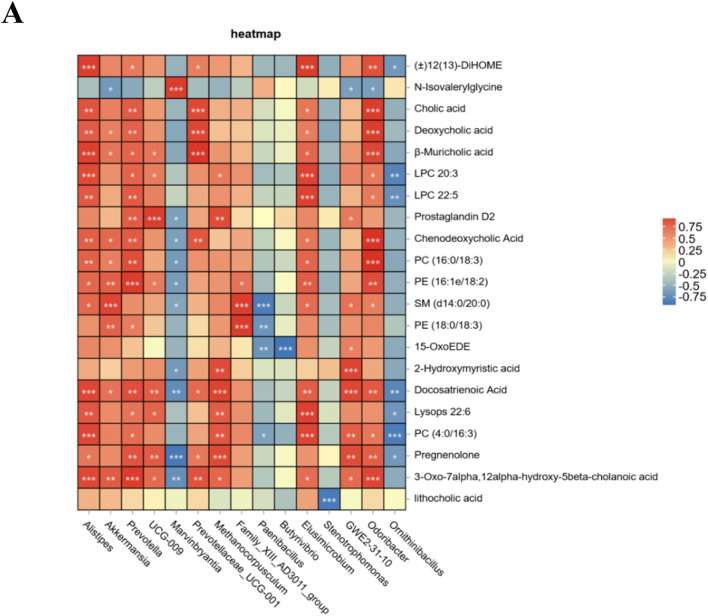
Association analysis of intestinal microbiota and serum metabolites in tea polysaccharide-fed beef cattle. **(A)** Spearman correlation analysis was conducted between the groups; red represents a positive correlation, and blue represents a negative correlation. *p < 0.05, **p < 0.01 and ***p < 0.001.

Finally, to investigate the relationship between the altered microbiota and serum differentially abundant metabolites, a Spearman correlation analysis was performed. The findings demonstrated that *Akkermansia, Alistipes, Prevotella, UCG-009, Prevotellaceae-UCG-001, Methanocorpusculum, Family_XIII_AD3011_group*, *Elusimicrobium*, and *GWE2-31-10* exhibited positive correlations with changes in serum metabolites. Furthermore, the tea polysaccharide group showed a significant increase in the abundance of *Akkermansia* and *Alistipes*. Conversely, *Marvinbryantia, Paenibacillus, Butyrivibrio, Stenotrophomonas*, and *Ornithinibacillus* were negatively correlated with changes in serum metabolites (Figure 6A). In conclusion, the consumption of tea polysaccharides resulted in a notable increase in the abundance of *Ackermania* and *Alistipes* in the intestinal microbiota of beef cattle. Moreover, the correlation analysis indicated a positive relationship between these bacteria and serum metabolites, suggesting that *Ackermania* and *Alistipes* may play a crucial role in mediating the physiological regulatory effects of tea polysaccharides on beef cattle.

## 4 Discussion

Long-term heat stress can lead to various detrimental effects in beef cattle, such as oxidative stress, inflammatory reactions, endocrine disorders, and immune dysfunction (Giannone et al., 2023). Due to its role as the primary metabolic and detoxification organ in animals, the liver is highly susceptible to damage caused by environmental heat stress. This vulnerability results in decreased production performance and disease resistance in cattle (Skibiel et al., 2018; Kop-Bozbay et al., 2021). The economic losses incurred by the beef cattle industry as a result of heat stress amount to a staggering 39.94 billion US dollars annually (Thornton et al., 2022). Consequently, it is of utmost importance to investigate the effects and mechanisms of heat stress on liver injury in beef cattle, as well as potential measures to mitigate these effects and enhance the overall performance and health of the animals.

The present study demonstrated the beneficial effects of tea polysaccharides, which are naturally occurring acidic glycoproteins, in reducing heat stress-induced food intake and hepatic inflammation. Furthermore, the expression of inflammatory marker genes such as NF-κB p65, TLR2, TLR4, RelB, TNF-α, and IL-1β was significantly decreased. By comparing the gut microbiota of cattle supplemented with or without tea polysaccharides, we found that supplementation increased the richness and diversity of gut bacteria in Simmental beef cattle. Notably, the relative abundances of beneficial bacteria such as *Akkermansia* and *Alistipes* increased, while the ratio of *Firmicutes* to *Bacteroidetes* and the abundance of *Agathobacter* decreased. These findings are significant, as recent studies suggest that *Akkermansia* and *Alistipes* play crucial roles in regulating fatty acid metabolism and inflammation. Furthermore, our study revealed that tea polysaccharides significantly increased the serum levels of stearic acid, linolenic acid, arachidonic acid, and adrenal acid, all of which have been proven to have anti-inflammatory effects. Additionally, our correlation analysis demonstrated a positive relationship between *Akkermansia*, *Alistipes*, and changes in serum metabolites, indicating that these bacteria may be pivotal in mediating the physiological regulatory functions of tea polysaccharides in beef cattle. Overall, this research suggested that supplementing tea polysaccharides can alleviate hepatic inflammation in cattle exposed to heat stress by modulating the composition of the gut microbiota in beef cattle.

As an acidic glycoprotein with natural biological activity found in tea leaves, tea polysaccharides, has been proved various physiological effects such as regulating immune function, inflammatory response and liver function in livestock recently Yuan et al. found that feeding tea polysaccharide could synergistically improve immune function and decrease the oxidative stress by enhancing the clearance of free radicals in mice (Yuan et al., 2015). It was also showeded that oral tea polysaccharides significantly decreased the levels of serum pro-inflammatory cytokines (TNF-α and IL-1β), and increased the expression levels of anti-inflammatory cells and immunoglobulin factors (IgA, IgG and G andIgM) in Kunming mice (Yang et al., 2012).Additonally, in broiler experiment, Li et al. denmonstrated that tea polysaccharide significantly increased contents of GSH-Px, SOD and T-AOC, and reduced content of MDA. It also increased Nrf2, NQO-1 and HO-1 mRNA expressions in meat of broiler (Li et al., 2022).These studies all indicate the important regulatory effects of tea polysaccharides on antioxidant, inflammatory and immune responses in the body.In the present study, we focused on the changes of NF-κB inflammatoy signaling pathway which consistent with the existing research reports. Otherwise, transcriptomic data showed that tea polysaccharide also activated other immune signaling pathway like Fc gamma receptor、B cell receptor、Fc epsilon RI and PPAR which were previous reported to alleviated the inflammatory response in various animal model (Wu et al., 2020; de Souza et al., 2021; Wahrmann et al., 2021; Kim et al., 2019; Fujimura et al., 2002). Similarly, tea polyphenols, another active natural component in tea, have been shown to regulate the expression of PPARγsignaling、Fc epsilon RI signaling and B cell receptor signaling pathway. For instance, it was reported that green tea polyphenol was significantly actived the PPARγ-responsive elements in endothelial cell (Fujimura et al., 2001). And Fujimura et al. found that tea polyphenol could suppress the activation of Fc epsilon RI pathway (Stefanie and Ruland, 2006; Liu et al., 2013). However, there are currently few reports on the regulatory mechanism of tea polysaccharides on Fc gamma receptor, B cell receptor, Fc epsilon RI and PPAR signaling pathway. Therefore, in addition to NF-κB, the study of hepatic inflammatory response of tea polysaccharides through other immune pathways is also worth further exploration in the future.

In our next research, further investigations are carried out to unravel the potential microflora mechanisms underlying effects of tea polysaccharides on hepatic inflammatory response. Previously, tea polysaccharides were shown to enhance immune function, reduce inflammation, and modulate glycolipid metabolism by influencing key species of the gut microbiota (Liu et al., 2018; Wang et al., 2023; Zeng et al., 2022; Yang et al., 2022). Research has revealed that feeding tea polysaccharides to mice with inflammatory bowel disease effectively improves the structure of their gut microbiota, inhibits the growth of harmful bacteria such as *Helicobacter pylori*, and increases the presence of beneficial bacteria such as *Akkermansia* and *Trichospira* (Zhang Y. et al., 2023). Li and Zeng’s study confirmed that tea polysaccharides can ameliorate obesity and related metabolic disorders in mice fed a high-fat diet by increasing the abundance of beneficial bacteria such as *Bacteroides*, *Bifidobacterium*, *Lactobacillus*, and *Akkermansia* (Li D. et al., 2023; Cristofori et al., 2021). Additionally, Zhu’s research reported that tea polysaccharides alleviate obesity, hyperlipidemia, and inflammation, improve intestinal barrier function, and mitigate dysbiosis of the gut microbiota in rats fed a high-fat diet by increasing the diversity of intestinal bacteria and the abundance of beneficial bacteria such as *Akkermansia*, *Lactobacillus*, and *Bacteroides* (Zhu et al., 2023). Interestingly, emerging evidence suggests that the *Akkermansia* and *Alistipes* genera play crucial roles in regulating inflammation in the body. *Akkermansia* and *Alistipes* exhibit significant negative correlations with inflammatory cytokines such as IL-6, IL12A, INF-γ, and TNF-α in humans, cattles, and mice (Bian et al., 2019; Zhao et al., 2023; Li et al., 2019). In our present study, we observed that tea polysaccharide intervention reduced the expression of hepatic inflammatory marker genes such as NF-κB p65, TLR2, TLR4, and RelB in cattle. The results of the bacterial community profile analysis demonstrated that tea polysaccharides significantly increased the abundance of *Akkermansia* and *Alistipes,* which is consistent with previous research findings. Overall, these results suggest that tea polysaccharides may alleviate heat stress-induced inflammation in cattle by regulating the abundance of inflammation-related gut bacteria.

Recently, there has been increasing recognition of the importance of the gut microbiota in influencing liver health in hosts. Previous studies have demonstrated that melatonin can reduce the expression level of the TLR4/NF-κB inflammatory signaling pathway in the liver of mice, ultimately alleviating aflatoxin-induced liver injury. This effect is achieved by decreasing the production of LPS derived from the gut and remodeling the intestinal microbiota (Liu et al., 2022c).

Kang et al. reported that high-fat diets lead to dysregulation of the structure of the intestinal microbiota, which in turn activates the TLR4/NF-κB signaling pathway in the liver (Kang et al., 2023). *Bacteroides* and *Firmicutes* are the two most common types of bacteria in the intestines. Studies have reported a significant increase in the ratio of *Firmicutes* to *Bacteroides* in liver diseases such as alcoholic liver and fatty liver (Aragon-Vela et al., 2021; Zhang et al., 2023c).

Furthermore, under heat stress, with increasing ambient temperature, the ratio of *Firmicutes* to *Bacteroidetes* in intestinal bacteria, as well as the serum inflammatory factors TNF-α, IL-6, and IL-1β, increases significantly in beef cattle (Yu et al., 2024). In the present study, feeding tea polysaccharide significantly increased the abundance of *Bacteroides* and alleviated the expression of the TLR4/NF-κB signaling pathway in liver inflammation. Moreover, heat stress decreased the abundance of *Firmicutes* and the ratio of *Firmicutes* to *Bacteroidetes* in the gut microbiota of beef cattle. These results further confirmed that tea polysaccharides regulate hepatic inflammation through the intestinal flora.

As vital organs in the body, the liver and intestine play crucial roles in each other’s physiological functions. In 1998, Marshall first proposed that the intestine and liver are interconnected through the portal vein system. Digestive tract metabolites and gut microbial products are absorbed into the liver via the superior mesenteric vein and inferior mesenteric vein, and this interaction is known as the “gut-liver axis” (Marshall, 1998). The gut microbiota primarily regulates inflammatory responses by producing short-chain fatty acids (SCFAs), such as butyric acid, propionic acid, and acetic acid. The bacteria responsible for producing SCFAs are considered to be the bridge between microbiota functions and epigenetic regulation, inflammatory mechanisms, markers, and personalized preventive healthcare (Tan et al., 2014). Extensive evidence has shown that *Akkermansia* is a well-known SCFA-producing bacteria in the microbiota (Li N. et al., 2023; Shen et al., 2023). In our study, after feeding tea polysaccharides, there was a significant increase in the abundance of *Akkermansia*, leading us to investigate the related metabolites in beef serum. Our metabolomics results demonstrated a significant increase in the serum levels of LCFAs, such as stearic acid, linolenic acid, arachidonic acid, and adrenic acid, in the tea polysaccharide feeding group. Although we did not observe significant changes in SCFA levels, previous studies have reported that LCFAs such as stearic acid (Kim et al., 2021; Pan et al., 2010), linolenic acid (Pauls et al., 2018; Saiki et al., 2017), arachidonic acid (Pereira et al., 2024; Roy et al., 2022), and adrenic acid (Brouwers et al., 2020) exert anti-inflammatory effects on the body. Currently, the reason for the inconsistent changes in SCFA and LCFA levels remains unclear, and further exploration is needed to understand the transformation pathway *in vivo*. Nonetheless, these results suggest that tea polysaccharides may influence LCFA levels in the cattle serum metabolome through the intestinal microbiota, thereby mediating the occurrence of hepatic inflammation.

Finally, the research provides a valuable insights into the potential benefits of tea polysaccharides in mitigating heat stress of beef cattle. However, there existed potential confounding variables like temperature, feed intake and body weight to influence the overall evaluation about the physiological function of tea polysaccharides. Therefore, the further exploration need to be carried out to demonstrate the thorough effects of tea polysaccharides on the body health of beef cattle in future studies. For instance, the effects of temperature and weight differences in the late fattening period on the results can be excluded by feeding tea polysaccharides in non-heat stress conditions and during the shelf cattle period.

In conclusion, our study indicated that long-term consumption of tea polysaccharides can mitigate heat stress-induced hepatic inflammation in cattle. Mechanistically, tea polysaccharides effectively improved the structure of the gut microbiota and the content of serum metabolites, suggesting a possible mechanism by which the liver-gut axis mitigates heat stress. These findings provide insight into the potential application of tea polysaccharides in alleviating heat stress in beef cattle.

## Data Availability

The original contributions presented in the study are publicly available. This data can be found here: NCBI https://www.ncbi.nlm.nih.gov/sra/?term=PRJNA1149939 and NCBI https://www.ncbi.nlm.nih.gov/sra/?term=PRJNA1149937.

## Supplementary material

Supplementary Figure S1 Influence of tea polysaccharides on food intake and hepatic transcriptome PCA distribution in beef cattle under heat stress. **(A)** Average daily food intake of beef cattle during the experiment. n = 6, **p < 0.01. **(B)** PCA plot of hepatic transcriptome in beef cattle fed with tea polysaccharides.
