# Supplementary figures and images for: Regulatory effects of tea polysaccharides on hepatic inflammation, gut microbiota dysbiosis, and serum metabolomic signatures in beef cattle under heat stress
Source: Front Physiol. 2024 Sep 6;15:1460414. doi: 10.3389/fphys.2024.1460414 (PMC11413490; doi:10.3389/fphys.2024.1460414)

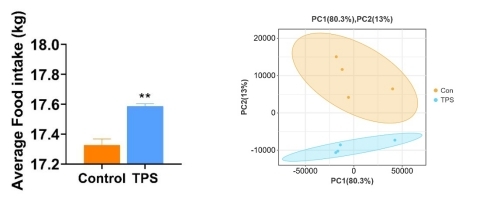

Supplement: Supplementary file 1 [file Image1.jpeg]
